# Important Sensory, Association, and Postprandial Perception Attributes Influencing Young Taiwanese Consumers’ Acceptance for Taiwanese Specialty Teas

**DOI:** 10.3390/foods9010100

**Published:** 2020-01-18

**Authors:** Bo-Kang Liou, Yih-Mon Jaw, George Chao-Chi Chuang, Newton N. J. Yau, Zhen-Yu Zhuang, Li-Fei Wang

**Affiliations:** 1 Department of Food Science and Technology, Central Taiwan University of Science and Technology, No. 666, Buzih Rd., Beitun Dist., Taichung City 40601, Taiwan; bkliou6755@ctust.edu.tw (B.-K.L.); 0956xxxxx5@gmail.com (Z.-Y.Z.); 2 Department of Chinese Culinary Arts, National Kaohsiung University of Hospitality and Tourism, No. 1, Songhe Rd., Xiaogang Dist., Kaohsiung City 81271, Taiwan; eamon@mail.nkuht.edu.tw; 3 Department of Food Science, China University of Science and Technology, No. 245, Academia Rd. Sec. 3, Nangang Dist., Taipei City 115, Taiwan; george0321@cc.cust.edu.tw; 4 Sinew Consulting Group Ltd., No. 26, Ln. 25, Shipin Rd., East Dist., Hsinchu City 300, Taiwan; newton.yau@e-sinew.com; 5 Hospitality and Tourism Research Center, National Kaohsiung University of Hospitality and Tourism, Kaohsiung City 81271, Taiwan

**Keywords:** CATA, sensory attributes, association attributes, postprandial perception attributes, teas (*Camellia sinensis*), degrees of fermentation

## Abstract

For hundreds of years, Taiwan has been famous for its various specialty teas. The sensory features of these teas have been well specialized and standardized through sensory evaluations performed by tea experts in yearly competitions throughout history. However, the question arises of whether young Taiwanese consumers, whose dietary behaviors have become Westernized, agree with the conventional sensory standards and association/postprandial concepts in the traditional tea market of Taiwan. To study young Taiwanese consumers’ ideas towards traditional specialty teas, this research recruited 109 respondents, younger than the age of 30, to taste seven Taiwanese specialty tea infusions of various degrees of fermentation, and their opinions were gathered by questionnaires composed of check-all-that-apply (CATA) questions and hedonic scales. Through statistical analyses, we found that several tea sensory attributes which were emphasized in experts’ descriptive sensory evaluations were not appreciated by the young Taiwanese people. Instead, tea aroma and late sweetness/palatable/smooth/refreshing mouthfeels were the most important sensory attributes contributing to their tea preference. Overall, there would generally be no problem in serving young Taiwanese consumers lightly-fermented oolong teas that generate the highest digestive and lowest heartburn postprandial perceptions.

## 1. Introduction

Based on processing methods and sensory qualities, teas (*Camellia sinensis*) can be divided into six types: green, yellow, dark, oolong, white, and black teas. Another kind of classification based on degrees of fermentation is more widely available in the global market and academic research and consists of unfermented green tea, semi-fermented oolong tea, and fully-fermented black tea [1], which account for 20%, 2%, and 78% of the total world tea production, respectively [2]. Thanks to the excellent natural environment, appropriate climate, and advanced agricultural techniques, Taiwan is one of the few areas/countries in the world where all types of tea can be simultaneously produced with the local raw materials, regardless of how the teas are defined or classified. Taiwan has long been superior and famous for its semi-fermented oolong teas. Since the 1860s, various oolong teas were exported from Taiwan to the world under the brand name “Formosa Oolong” [3]. In 2018, Taiwan exported about 8000 tons of tea which cost 111.9 million United States Dollars (USD). Based on data from 2014 to 2018, Taiwan was also the fastest-growing tea exporter in the world [4].

In 1903 the government-supported Tea Research and Extension Station (TRES) was established to promote Taiwanese teas. Until now, the research work of this institute was never suspended, even as the political environment of Taiwan has changed. In addition to cultivating new tea species, which usually takes decades, TRES also instructs farmers on how to process high-quality teas. The conditions of tea processing are not supposed to remain immutable because of the inconstant weather and natural environment. Therefore yearly, TRES holds competitions for various Taiwanese specialty teas to encourage farmers to enhance and create new tea processing techniques. As a result of the high credibility of TRES’s tea competitions in Taiwanese society, it is a great honor for farmers if their techniques/products are recognized in these competitions. Taiwanese consumers are also proud of having the champion teas even though their prices could raise ten times.

Throughout the long history of annual competitions in which TRES authorities appraise the teas by sensory evaluation, the qualities of various Taiwanese specialty teas have been well standardized. Assisted by modern chemical analyses, standardization can now be quantified. However, all of these sensory standards were established by tea experts, and until now, little information had been collected directly by consumer investigation. Nowadays, Taiwanese young generations are very much Westernized in their dietary behaviors and choices, so there arises the question of how young people accept conventional tea products. How much do they agree with the present sensory standards applied to specialty teas? Are traditional advertising words/terms used in Taiwanese tea sales still attractive or convincing for young people? Is it necessary to modify marketing strategies to strive for more young customers? To study young Taiwanese consumers’ ideas for traditional specialty teas, this research recruited respondents younger than 30 years old to evaluate tea infusions, and their opinions towards the sensory qualities of teas and their associations and postprandial perceptions elicited by tea drinking were gathered through questionnaires composed of check-all-that-apply (CATA) questions and hedonic scales.

## 2. Materials and Methods

### 2.1. Tea Samples

Seven Taiwanese specialty teas of various degrees of fermentation were studied. Bi-Luo-Chun (BLC) is an unfermented green tea produced in the Sanxia District of northern Taiwan. Different from the Japanese Sencha whose fresh tea leaves are fixed by steaming, BLC, similar to most Chinese green teas, is fixed by pan-frying which has been mostly replaced by baking in rolling drums in modern processing. Wenshan Pouchong tea (WSP) produced in the Pinglin District of northern Taiwan is the most lightly fermented oolong tea. Different from other oolong teas which are crumpled into a ball shape to damage the tea leaf structure for effective brewing, WSP is not kneaded so that it presents a striped shape. High-mountain tea (HMT), also called Alpine oolong, is another lightly-fermented oolong tea made of tea leaves harvested from mountains higher than 1000 m above the sea level. In high-elevation regions with lower temperatures and less sunshine, less bitter and astringent compounds, such as caffeine and catechins, are synthesized in the fresh tea leaves [5] and processed by heavy withering followed by light fermentation. Dong Ding oolong tea’s (DDO) special sensory qualities are attributed to the dense fog of its origin, the Dong Ding mountains (altitude 500~800 m) in the middle of Taiwan. Its fermentation has been defined as “medium degree” which is characterized by the detectability of methyl salicylate that continuously increases as the fermentation degree is enhanced [6]. Iron Goddess Tea (Chinese call it “Tie Guanyin”, TGY) is another oolong tea processed with heavier fermentation than DDO. Different from the Anxi green Iron Goddess produced in Fujian, China which is not roasted and green in character, Taiwanese TGY is known in the tea market for its delicate roasting techniques. Baihao oolong (BHO), also named Oriental Beauty, is the most heavily fermented oolong tea. Its raw material is young shoots with white floss, to which pesticides should not be used, infested by *Jacobiasca formosana* (a type of green fly). The injured tea leaves are said to give a “honey-like” flavor after fermentation [6]. Taiwan also produces several kinds of fully-fermented black teas. Yuchi Township of Nantou County in the middle of Taiwan has been famous for its black tea since the early 1900s. In addition, the Assam cultivar that was planted during the Japanese colonial period and Taiwanese local species (e.g., Tai-Cha No. 8, 12, 18, and 21) have also been cultivated for black tea production by TRES of the Taiwanese government. In this study, the Yuchi Sun-Moon-Lake black tea (SML) manufactured from the variety Tai-Cha No. 18 was adopted as the representative sample of Taiwanese black teas. In Table 1, characteristics of these seven Taiwanese specialty teas are summarized [7]. 

For sensory evaluation, all of the tea leaves purchased were similarly at a moderate level of quality as distinguished by their unit prices. The tea infusion samples were prepared according to the standard method of ISO 3103:1980. All of the tea leaves were individually prepared into tea infusions by brewing with 50 times of boiling distilled water (e.g., brewing 3 g tea leaves with 150 mL boiling water). After letting stand in the vessels covered with lids for 5 min, the tea leaves were quickly removed with strainers, then the tea infusions were loaded into thermoses and served to the respondents. Only tea infusions freshly prepared within 30 min were used for this study.

### 2.2. Preliminary Studies and Questionnaire Design

Originally, 175 descriptive terms regarding color, aroma, flavor, mouthfeel, and aftertaste of tea infusions were collected from 24 English and Chinese tea research papers [8,9,10]. Tea infusion samples described above were firstly tasted by four of our sensory researchers, and then 54 sensory attributes were selected according to the researchers’ consensus (Table 2). Among them, “late sweetness” and “late fragrance” were defined as the mouthfeels that might not be perceived when the samples just entered people’s mouths. As time passed and before the samples were swallowed, people might gradually feel the formation of sweetness or fragrance in their oral cavities. Contrary to the mouthfeels that express the perceptions before the samples were swallowed, aftertaste was the residuals that still remained after the samples were swallowed.

Also, a tea marketing study was conducted to search for the terms frequently used to describe the associations and postprandial perceptions of tea merchandises in the Taiwanese market (Table 3). In Taiwan, tea products are often promoted with cultural activities, such as contests of brewing tea techniques, brewing teas with art tea sets, tea tasting with art appreciation, or matching teas with culinary creations. Therefore, drinking teas may be associated in Taiwanese customers’ minds with special cultural concepts. During the test, all respondents were blind to the appearance and prices of the original tea leaves, but they were requested to surmise the tea prices (expensive or cheap) by tasting the tea infusions. If they had felt that the tea qualities were higher than the prices they speculated, then the item “high CP (cost-performance ratio) value” should be checked. In addition, digestif, which is understood as cutting the greasy/sweet cloying of rich foods in Taiwanese language, is a term used in the conventional tea market of Taiwan for long and may be better comprehended by Taiwanese consumers than the other health benefits of teas demonstrated by the modern researches, such as antioxidant, anti-hyperglycemic, or anti-cancer activities [2]. Conversely, quite a few people do not drink teas owing to their unhealthy stomachs. Even though the causes are not clear, various intensities of heartburn symptom indeed appear in some people after drinking teas [11]. A recent research revealed that caffeine could be one of the tea compounds to induce gastric acid secretion via activating the bitter taste receptors in oral cavities and stomachs [12].

In addition to the CATA questions consisting of checklists of sensory, association, and postprandial perception attributes, a 9-point hedonic scale was also incorporated into the questionnaire to investigate consumers’ overall acceptability of the tea samples. As defined by Peryam and Pilgrim, scores 1 to 9 were explained in order as “dislike extremely” (score 1), “dislike very much” (score 2), “dislike moderately” (score 3), “dislike slightly” (score 4), “neither like nor dislike” (score 5), “like slightly” (score 6), “like moderately” (score 7), “like very much” (score 8), and “like extremely” (score 9) [13].

### 2.3. Procedure of Sensory Evaluation

According to Ares et al., 60–80 consumers can be regarded as a reasonable compromise to get a stable sample and descriptor configurations when working with widely different samples [14]. In addition, International Organization for Standardization (ISO 11136:2014) recommended that at least 80–100 participants are required to conduct hedonic tests with consumers in a controlled area. Therefore, this evaluation proceeded for 4 consecutive working days until 109 effective responses were collected from 54 male and 55 female volunteers. Most of the participants were the staff and students of Central Taiwan University of Science and Technology (CTUST). A few off-campus subjects were also invited. The qualified subjects were aged between 18 and 30 years old and experienced in drinking all of seven Taiwanese teas being studied.

After a short explanation of the operation, the qualified subjects were guided to a professional sensory evaluation room with semi-closed booths equipped with white light and computers (Compusense Five^TM^) through which the questions were answered. All of the tea infusion samples were marked by random three-digit numbers and presented to the respondents by a sequential monadic technique with William Latin square design [15]. At the same time, the individual respondents were requested to taste seven tea infusion samples and check 18 questions of 4 categories for each sample. Before tasting a new sample, respondents were instructed to clean their palates with crackers and pure water. Thirty seconds after all questions for one sample were answered, another empty questionnaire with the same checklists would be shown to the respondents for the next sample. The respondents were not allowed to modify their answers for the previous samples once the questionnaires were renewed.

### 2.4. Statistical Analysis

XLSTAT [16] was used for the following statistical analyses. Cochran’s Q test was used to compare the frequencies of attribute selection from the CATA questions, and post hoc analysis was conducted using multiple pairwise comparisons with the McNemar test (Bonferroni). The correspondence analysis with Chi-squared distance was calculated on the attributes responded by more than 20% of participants to measure similarities among the tea infusions. Consumers’ overall liking of the tea infusions averaged from the 9-point hedonic scores was analyzed by one-way ANOVA in conjunction with Tukey’s honestly significant difference (HSD) test. Agglomerative hierarchical clustering analysis with Ward’s method and automatic truncation based on entropy were applied to classify the respondents according to their homogeneous overall liking for tea consumption. Finally, partial least squares regression (PLSR) analysis between the hedonic scores and the CATA data was performed to understand how the sensory, association, and postprandial perception attributes impacted respondents’ hedonic judgments.

## 3. Results and Discussion 

The conventional sensory evaluation of TRES is usually performed by a few tea experts whose level of attainment demands strict and long-term training. Even though several alternatives, such as open-ended questions or a long series of pre-determined intensity scales, have been tried to gather customers’ direct opinions, these methods were not only laborious but also often returned a lack of consensus and limited insight. In recent years, CATA investigation has been getting popular in consumer research due to its apparent simplicity in technique. CATA respondents are presented with a pre-determined list of terms but only have to tick those which they think applicable to the test samples, and no intensity consideration is required [17,18]. The provision of a list of words can help those respondents who have difficulty in expressing perception, and the terms used in CATA lists can not only be purely sensory, but also emotional or functional [19]. In the end, the CATA data are interpreted by statistical analyses to characterize/differentiate the samples.

### 3.1. Differences of Sensory Attributes Among the Taiwanese Specialty Tea Infusions

As shown in Table 2, the participants perceived the 54 attributes of five sensory dimensions (appearance, aroma, flavor, mouthfeel, and aftertaste) for tea infusions in various frequencies. The *p*-values calculated by Cochran’s Q test indicate that 41 sensory attributes were perceived to have a significant difference among the seven tea infusions.

In Table 2, the seven tea samples were listed from left to right in order of increased fermentation degrees. According to the structure of bar charts which expressed the proportions (%) of respondents’ selection, it was determined that the changes of sensory attributes caused by fermentation did not proceed in a gradual style. On the contrary, the organoleptic properties of these seven teas were clearly discriminated at the point between DDO and TGY. The lightly-fermented oolong teas, including WSP, HMT, and DDO, possessed sensory qualities similar to those of unfermented BLC green tea. In contrast, TGY and BHO, the oolong teas with heavier degrees of fermentation than DDO, which was recognized as the index of medium fermentation [6], displayed sensory properties close to those of fully-fermented SML black tea. This revealed that some of the compounds that caused the variation of sensory qualities could not be synthesized or perceptible unless the degree of fermentation achieved a certain level. For example, it is known that theaflavins and thearubigins, the condensed products of catechins and their gallates by enzymatic oxidation, are the major compounds contributing to the color and taste of black tea. Theaflavins are the dimer catechins with the benzotropolone structure showing an orange-red color. Prolonged fermentation may result in the formation of thearubigins with the structures of more polymeric degrees, which are red-brown in color [20,21]. As shown in Table 2, the amber (defined as yellowish-orange), orange-red, yellow-brown, and brown colors are visually perceptible only in the infusions of heavily-fermented oolong teas and black tea. By contrast, catechins are one of the major compounds contributing to the astringency and bitterness flavors of green tea [8]. Oxidation of catechins occurring in fermentation could lower the intensities of astringency and bitterness in the lightly-fermented oolong teas. However, as fermentation was continued, formation of theaflavins and thearubigins significantly enhanced the astringency and bitterness levels of BHO and SML [22,23,24]. Even though the formation of theaflavins/thearubigins in TGY was visually detectable, the high-temperature roasting in its final processing may induce some chemical reactions, such as polymerization [25] or carbonization [26], which could decrease the soluble solids of TGY and lower its astringency and bitterness to levels comparable with those of lightly-fermented oolong teas. In addition, a significantly stronger honey aroma could be perceived in the heavily-fermented teas (BHO and SML). Phenylacetaldehyde, with an odor threshold of 4 ppb and derived from the amino acid phenylalanine was reported as one of the major sources of the honey-like aroma occurring in the fermented teas [27,28]. Fermentation was also shown to enhance the fresh aroma and refreshing taste of teas (WSP, HMT, and DDT) which could be masked by the roasted flavor as a stronger roasting processing usually accompanies heavily-fermented teas (TGY, BHO, and SML). It is worth noting that the fully-fermented SML displayed the highest pungency which could result in its lowest mildness and palatable mouthfeels.

**Figure 1:**
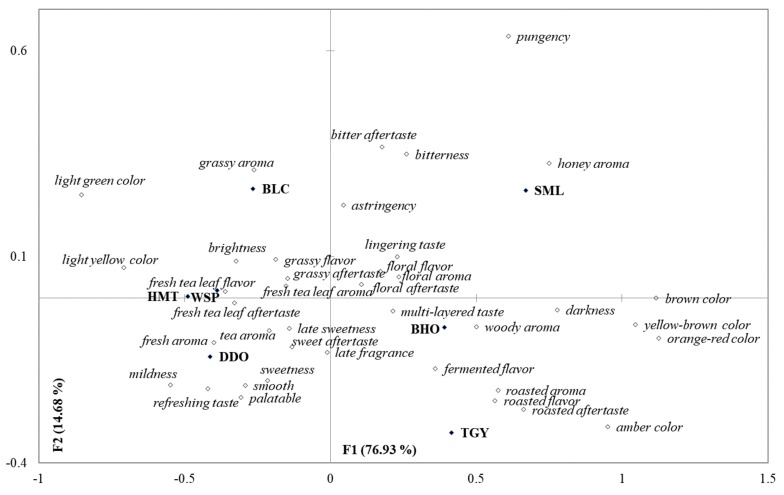
A bi-plot by correspondence analysis of the seven tea infusions in association with the 39 sensory attributes selected by more than 20% of participants. BLC: Bi-Luo-Chun green tea, WSP: Wenshan Pouchong tea, HMT: High-mountain oolong tea, DDO: Dong Ding oolong tea, TGY: Iron Goddess oolong tea, BHO: Baihao oolong tea, SML: Yuchi Sun-Moon-Lake black tea.

The 39 terms marked with asterisks (*) were the attributes perceived by more than 20% of respondents. According to the Pareto principle, these 39 sensory attributes could be recognized as important characteristics determining the sensory qualities of Taiwanese teas, while the other 15 attributes would be considered irrelevant [29,30]. With the 15 irrelevant items ignored, the data of the 39 judgmental attributes were further processed by correspondence analysis with Chi-squared distance calculation to measure the similarities of these tea samples. As shown in Figure 1, the unfermented BLC and lightly-fermented WSP, HTM, and DDO were clearly differentiated from the heavily-fermented TGY, BHO, and SML based on Factor 1 (76.93%). Through Factor 2 (14.68%), the heavily semi-fermented TGY and BHO were further divided from the fully-fermented SML. Compared to the semi-fermented oolong teas, the fully-fermented black tea and unfermented green tea displayed stronger bitterness/astringency flavors, pungency mouthfeel, and bitter aftertaste. Among the oolong teas, the lightly-fermented ones (WSP, HMT, and DDO) were perceived as being higher in fresh tea leaf aroma/flavor/aftertaste, fresh/tea aromas, and sweetness flavor/aftertaste. They were also perceived to have the higher mouthfeels of palatable, late sweetness, smooth, refreshing, and mildness than the other teas.

### 3.2. Young Taiwanese Consumers’ Associations Originated from the Taiwanese Specialty Teas

To avoid confusion with the sensory attributes, the association and postprandial perception attributes in the following discussion are written with capital letters. Among the 10 association attributes listed in Table 3, two of them, “Imported” and “Expensive”, were discerned by less than 20% of the respondents. Probably all these 7 tea products frequently appeared in the respondents’ daily life and their sensory characteristics were familiar to the respondents, so they were not considered “Imported” and “Expensive”. On the contrary, “common items” could be easily misunderstood as the products of “low in price (Cheap)”. However, more than 20% of respondents agreed that all of these tea samples possessed a “High CP value” for which respondents did not perceive a significant difference amongst the 7 tea infusions (*p* > 0.05). This revealed that all of the tea samples brought consumers the similar sense of value, and “price” might not be an influential factor for the respondents’ judgements on the other attributes.

**Figure 2:**
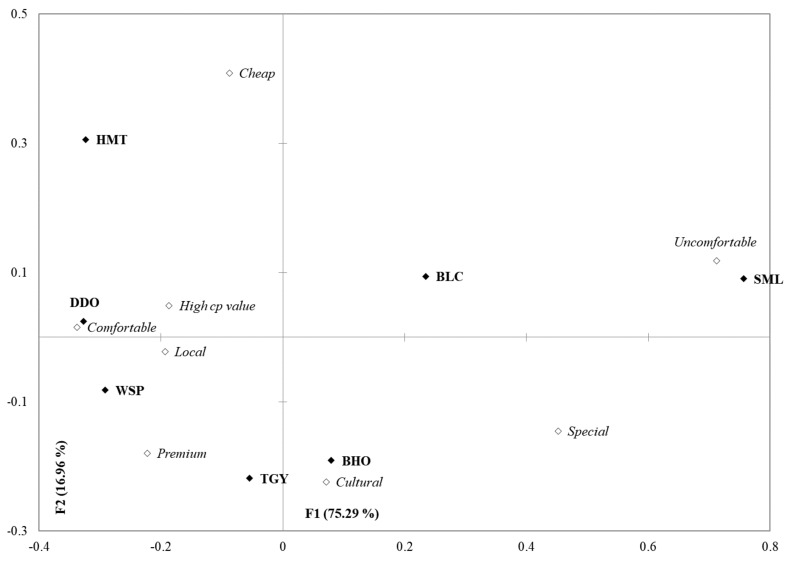
A bi-plot by correspondence analysis of the 7 tea infusions in association with the 8 association attributes selected by more than 20% of participants. BLC: Bi-Luo-Chun green tea, WSP: Wenshan Pouchong tea, HMT: High-mountain oolong tea, DDO: Dong Ding oolong tea, TGY: Iron Goddess oolong tea, BHO: Baihao oolong tea and SML: Yuchi Sun-Moon-Lake black tea. “High CP (cost-performance ratio) value” could be checked when consumers felt that the tea qualities were higher than the prices they speculated.

Figure 2 shows the correspondence analysis of the 8 association attributes selected by more than 20% of respondents. On Figure 2, the attributes “Comfortable” and “Uncomfortable” were located at almost the both extremes of Factor 1 (75.29%). Proper level of fermentation processing (semi-fermentation) significantly enhanced the Comfortable association of teas. However, over fermentation could reduce the Comfortable level of fully-fermented SML to even more Uncomfortable than the unfermented BLC. Different from the other tea markets where oolong teas are usually the minor species or even cannot be found, Taiwan is one of the few oolong tea origins in the world. For Taiwanese consumers, the globally widespread black and green teas seemed more “Special” than the oolong teas that were available at their fingertips. Even though oolong teas are the common products in Taiwanese’ daily life, many of them were still appreciated for their “Premium” (high quality) by the young Taiwanese consumers. In Figure 2, the heavily semi-fermented BHO and TGY with the profound/rich sensory qualities, as expressed by the overlapping area of positive Factor 1 and negative Factor 2 in Figure 1, were also shown to leave the deepest “Cultural” impression in young Taiwanese consumers’ minds. 

### 3.3. The Postprandial Perceptions Elicited in Young Taiwanese Consumers by Tasting the Taiwanese Specialty Tea Infusions

The 10 postprandial perception attributes listed on Table 3 were the commercial languages adopted from Taiwanese tea market. The respondents seemed familiar with these terms, and all of them were ticked by more than 20% of answerers. “Single blend” is used in Taiwanese market to guarantee the genuineness of tea products that are not blended with the lower-graded or non-local tea leaves. In addition, “salivary stimulation” and “thirst quenching” are usually used together as a combined advertising term for beverage products in Taiwan. Even though “salivary stimulation” was not perceived as an important mouthfeel when tea infusions were still remained in respondents’ mouths (Table 2), “Thirst quenching” was eventually recognized as an enjoyable postprandial perception by consumers (Table 3).

**Figure 3:**
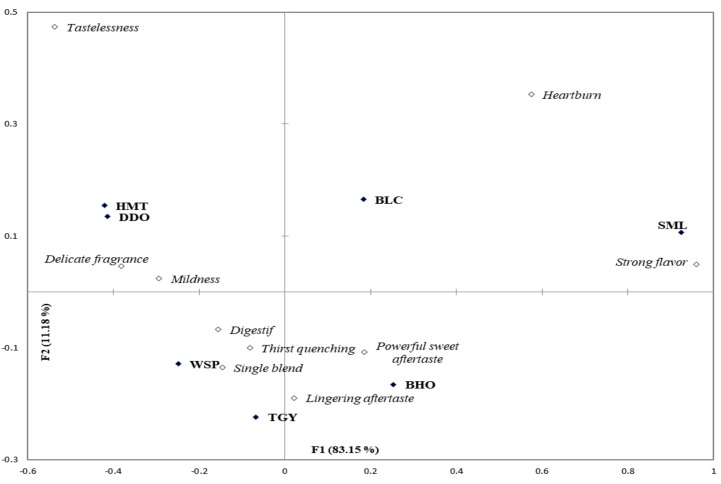
A bi-plot by correspondence analysis of the 7 tea infusions in association with the 10 postprandial perception attributes selected by more than 20% of participants. BLC: Bi-Luo-Chun green tea, WSP: Wenshan Pouchong tea, HMT: High-mountain oolong tea, DDO: Dong Ding oolong tea, TGY: Iron Goddess oolong tea, BHO: Baihao oolong tea and SML: Yuchi Sun-Moon-Lake black tea.

Since long, “Heartburn” has been tabooing quite a few customers from drinking teas, and “mild with no cause of heartburn” is one of the important advertising languages for Taiwanese merchants to publicize their tea products. Actually, there have been no consistent results obtained from the researches on the association between tea consumption and gastroesophageal reflux disease (GERD). Even though caffeine and theobromine have been suspected as some of the ingredients for heartburn, so far no clinical trials have been performed to definitively demonstrate that removing such foods from diet leads to the relief of GERD symptom [31]. Some of the newest researches even concluded that there was no significant relationship between tea consumption and the risk of GERD [32,33]. However, our CATA data revealed that some respondents indeed perceived certain level of “Heartburn” (stomach uncomfortableness) after drinking some of the tea infusions, and the fully-fermented SML and unfermented BLC were positioned closest to “Heartburn” on Figure 3. 

As a timeless beverage whose popularity has never decreased in Taiwan for hundreds of years, the sensory characters of teas should act well in concert with those of Taiwanese foods. Different from the English habit of afternoon tea, Taiwanese people usually have teas after meals for “Digestif”. On our questionnaire, this term “Digestif” was expressed as “cutting the redundant grease/sweetness of rich foods”. Even though it is prohibited by modern food law to claim “functions” on general foods, it is a deeply rooted Taiwanese concept and belief that drinking teas can help remove fat/sugar. In Figure 3, the semi-fermented oolong teas were perceived stronger postprandial “Digestif” than the unfermented and fully-fermented teas. Also, the lightly-fermented oolong teas brought consumers more sensations of “Delicate fragrance” and “Mildness”. Even though “Tastelessness” is sometimes a negative attribute in the tea market and is often confused with “Mildness” by untrained respondents, “Tastelessness” was positioned much farther than “Mildness” from the lightly-fermented oolong teas in Figure 3. In the Taiwanese tea market, “Strong flavor” is a neutral term which represents neither the “positive” nor “negative” character of teas. However, the young respondents placed it at the entirely opposite extreme of “Delicate fragrance” based on Factor 1 (81.21%) of correspondence analysis, and the fully-fermented SML possessed the highest postprandial feature of “Strong flavor” (Figure 3). 

### 3.4. Young Taiwanese Consumers’ Overall Preference for the Teas of Various Fermentation Degrees 

Based on the 9-point hedonic test, young Taiwanese consumers’ acceptance for various Taiwanese tea infusions is presented in Table 4. As shown in the column of “overall liking”, young Taiwanese consumers accepted these tea samples between the levels of “dislike slightly” (score 4) and “like moderately” (score 7), and they preferred the semi-fermented teas rather than the unfermented and fully-fermented ones (*p* < 0.05). Even though the respondents liked various oolong teas at a similar level, the most lightly-fermented WSP was significantly more preferred to the most heavily-fermented BHO (*p* < 0.05). 

Through hierarchical clustering analysis, the respondents were classified into three types of tea consumers; Cluster 1, 2, and 3 accounted for 33.9%, 45.0%, and 21.1% of the total respondents, respectively. Overall, the consumers of Cluster 1 and Cluster 2 liked the Taiwanese specialty teas much more than Cluster 3, except DDO was equally preferred by all three clusters. The consumers of Cluster 1 (33.9%) accepted all of these teas at the level between “neither like nor dislike” and “like moderately”, and there was no significant difference among their preference mean values for these seven tea samples (*p* > 0.05). This group of young consumers displayed high acceptance for all kinds of teas, and was defined as consumers “enjoying teas no matter what kind”. The consumers of Cluster 2 (45.0%) took all of the teas at the level between “dislike moderately” and “like moderately”, and they significantly preferred all oolong teas to green and black teas. This group of people was considered as “oolong tea lovers”. The consumers of Cluster 3 (21.1%) accepted Taiwanese specialty teas at the level between “dislike very much” and “like slightly”, and they significantly preferred the lightly-fermented oolong teas to the others. They especially could not accept the fully-fermented SML and unfermented BLC. This group of consumers was classified as “lightly-fermented oolong tea lovers”. As a comprehensive outcome, it could be recommended that there would generally be no problem in serving young Taiwanese consumers lightly-fermented oolong teas.

### 3.5. Impact of Various Tea Attributes on Young Taiwanese Consumers’ Hedonic Judgements by Partial Least Squares Regression (PLSR) Analysis

**Figure 4:**
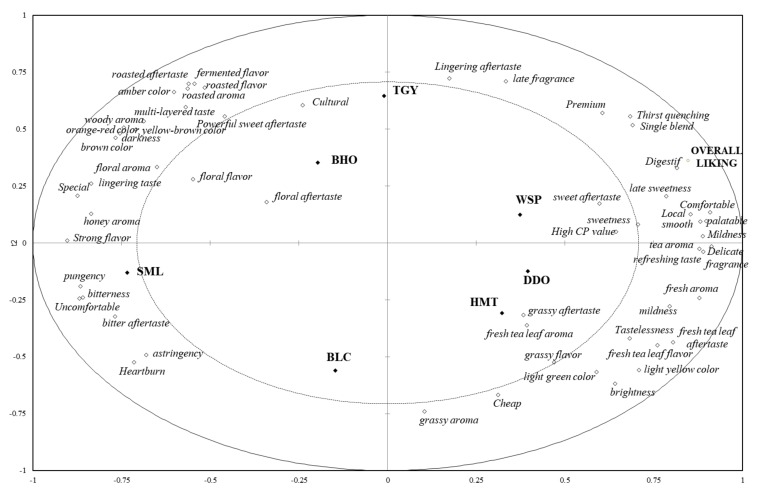
The partial least squares regression (PLSR) correlation loading plot of sensory, association, postprandial perception attributes and overall liking of seven Taiwanese specialty teas. The model was derived from attribute data as the X-matrix and overall liking data as the Y-matrix. The default values for radii correspond to 50% and 100% explained variance, respectively. BLC: Bi-Luo-Chun green tea, WSP: Wenshan Pouchong tea, HMT: High-mountain oolong tea, DDO: Dong Ding oolong tea, TGY: Iron Goddess oolong tea, BHO: Baihao oolong tea, SML: Yuchi Sun-Moon-Lake black tea. “High CP (cost-performance ratio) value” was checked when consumers felt that the tea qualities were higher than the prices they speculated.

As shown in Figure 4, respondents’ selection frequencies of various sensory, association, and postprandial perception attributes and overall liking judgements for seven Taiwanese specialty teas were analyzed by partial least squares regression (PLSR) and expressed as a correlation loading plot. The model was derived from attribute CATA data as the X-matrix and overall liking scores as the Y-matrix. The attributes between the two circles were the more important factors, while the variables located inside the inner ellipse were less influential for young Taiwanese people’s judgements of tea liking [34]. Some of them, such as sweet aftertaste, grassy aftertaste/flavor, and fresh tea leaf aroma, were attributes showing no significant difference among the tea samples (Table 2). In addition to fruity flavor/aftertaste which were excluded from the relevant attributes according to the Pareto principle [29,30], PLSR analysis also revealed that floral flavor/aftertaste were not important characters for young Taiwanese consumers to choose tea products (Figure 4). Actually, “floral” and “fruity” are recognized as two of the major positive natures for teas based on the TRES standards and many descriptive sensory evaluation researches fulfilled by trained sensory panelists [9]. Unexpectedly, our results show that many young Taiwanese consumers could not perceive fruity aroma/flavor from the tea infusions, and floral flavor/aftertaste also did not play an important role in their tea judgements. It indicated a significant difference in descriptor cognition/comprehension between trained evaluators and general consumers, and suggested that sometimes sensory evaluation performed directly by consumers could obtain results closer to market authenticity. 

The spot “overall liking” is located on the upper right portion of Figure 4. Compared to the other teas, the three lightly-fermented oolong teas were closer to the “overall liking” spot, and WSP was the most preferred by young Taiwanese consumers. The top five sensory attributes determining young Taiwanese people’s liking, that is, the five sensory attributes closest to the “overall liking” spot on Figure 4, were tea aroma and late sweetness/smooth/palatable/refreshing mouthfeels. Contrarily, the items located on the opposite side of the “overall liking” spot, such as pungency mouthfeel, bitterness/astringency flavors, and bitter aftertaste on the lower left corner were the major sensory attributes hindering young Taiwanese people’s acceptance for a tea product. It was interesting to find that “honey aroma”, which used to be commended for many tea products, seemed not to be appreciated by young Taiwanese people. In addition, “Comfortable” and “Local” were the most important positive associations occurring in the respondents’ replies. In view of tea products, young Taiwanese consumers seemed satisfied with the local products which also generated for them a “Premium” concept. The young consumers would not pursue “Special” tea products, because they felt more “Comfortable” with the “Local” flavors. Furthermore, “Digestif”, “Single blend”, “Thirst quenching”, “Mildness”, and “Delicate fragrance” were the important postprandial perceptions that made young Taiwanese consumers like a tea product. Overall, “Digestif” was the most important attribute. Even though the anti-obesity effect of green/black teas is being proven by more and more high-fat/high-sucrose-driven metabolism researches [35,36,37,38], the refreshing mouthfeel resulting from cleaning the greasy/sweet cloying in oral cavities by lightly-fermented oolong teas might be the more direct source of “Digestif” sensation perceived by Taiwanese consumers (Figure 4). Regarding the correlation between “Digestif” perception and real physiological metabolism, it may be interesting to conduct further in vitro/in vivo experiments to demonstrate if lightly-fermented oolong teas perform a higher anti-obesity effect than others. On the other hand, “Heartburn” was the major postprandial perception impeding young Taiwanese consumers’ preference of tea products. Consumers’ perception of heartburn level seemed in line with the bitterness, astringency, and pungency intensities of teas, and was also closely related to “Uncomfortable” association (Figure 4).

## 4. Conclusions

Through CATA sensory evaluation coupled with hedonic tests, this research efficiently disclosed the most important sensory, association, and postprandial perception attributes impacting the young generation’s choice of tea products in Taiwan. Several tea sensory attributes that were emphasized in experts’ descriptive sensory evaluations, such as fruity/floral flavors, sweet aftertaste, and honey aroma, were actually not admired by the young Taiwanese consumers. Instead, late sweetness/palatable/smooth/refreshing mouthfeels and tea aroma were the most important five sensory attributes contributing to their tea preference, and these sensory attributes were significantly more perceivable in the lightly-fermented oolong teas. Based on PLSR analysis, “Digestif” was the most powerful attribute that enhanced young Taiwanese people’s preference for a tea product, while “Heartburn” was more frequently accompanied with drinking fully-fermented and unfermented teas; this might be the most negative postprandial perception lowering young Taiwanese people’s acceptance of teas. The SML black tea with the strongest bitterness/astringency flavors, pungency mouthfeel, and bitter aftertaste also generated the highest “Uncomfortable” association and “Strong flavor” postprandial perceptions which made it the least acceptable to the young Taiwanese consumers. Overall, it was revealed by hierarchical clustering analysis of the hedonic data that there would generally be no problem in serving young Taiwanese people lightly-fermented oolong teas.

Customers from various backgrounds usually have various requirements for different products. This research demonstrated that changes and improvement are always necessary, even for products that have been traditionally highly recognized, because people’s preference may vary alongside lifestyle changes. CATA analysis coupled with hedonic tests are efficient tools for the research of various products, customers, and markets. Compared to experts’ opinions, information directly from consumers may be closer to real market requirements. 

## Figures and Tables

**Table 1:** The seven representative Taiwanese teas used in this research [7].

Full Name of Tea	Abbreviation	Classification (Appearance of Dry Tea Leaves)	Fermentation Degree (%)	Origin
Bi-Luo-Chun tea	BLC	Green tea (bright green/curly)	0	New Taipei City
Wenshan Pouchong tea	WSP	Oolong tea (rich green/curly)	12–15	New Taipei City
High-mountain tea	HMT	Oolong tea (rich green/hemispherical)	20–30	Chiayi County
Dong Ding tea	DDO	Oolong tea (rich green/hemispherical)	20–30	Nantou County
Iron Goddess tea	TGY	Oolong tea (deep green/spherical)	30–40	Taipei City
Baihao tea	BHO	Oolong tea (multi-hued/spherical)	60–70	Hsinchu County
Yuchi Sun-Moon-Lake tea	SML	Black tea (dark brown/stretched or twisted)	>90	Nantou County

**Table 2:**
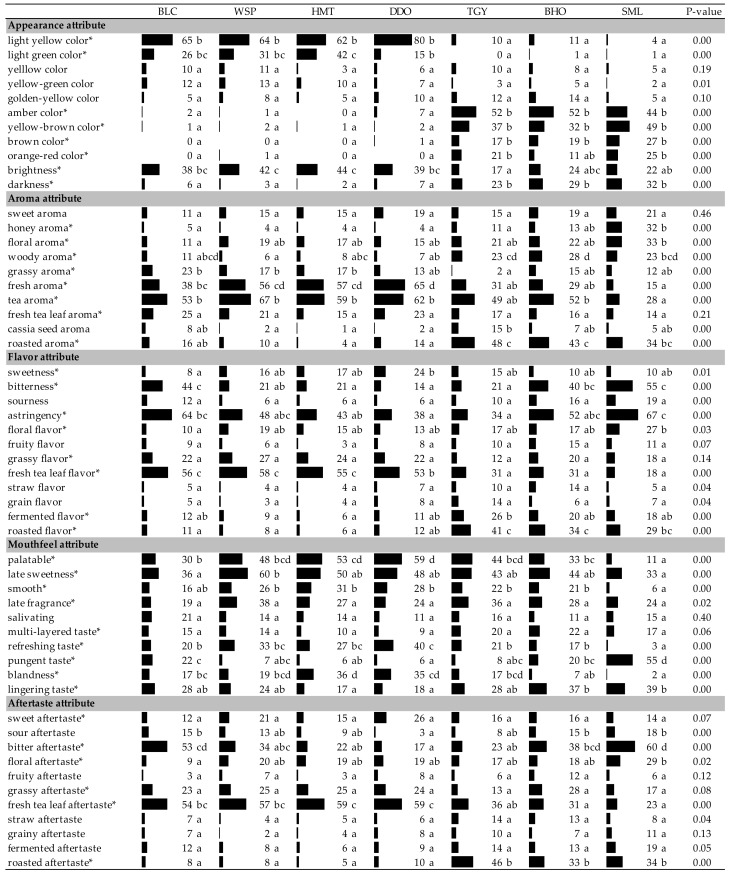
Selection frequencies of sensory attributes for the Taiwanese specialty tea infusions based on the check-all-that-apply (CATA) method (*n* = 109).

Significant difference (*p* < 0.05) of panelists’ selection proportions for each attribute among the seven tea samples were determined by Cochran’s Q test. The pairwise post-hoc McNemar test (Bonferroni) was also performed, and the tea samples signed with different alphabet letters were significantly different in the frequencies of panelists’ selection for the attribute listed in the same row. Asterisks are marked on the attributes selected by more than 20% of respondents. BLC: Bi-Luo-Chun green tea, WSP: Wenshan Pouchong tea, HMT: High-mountain oolong tea, DDO: Dong Ding oolong tea, TGY: Iron Goddess oolong tea, BHO: Baihao oolong tea, SML: Yuchi Sun-Moon-Lake black tea.

**Table 3:**
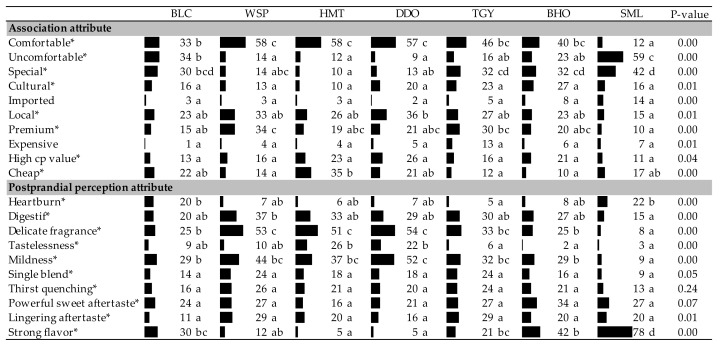
Participants’ selection frequencies of association and postprandial perception attributes for the Taiwanese specialty tea infusions based on CATA method (*n* = 109).

Significant difference (*p* < 0.05) of panelists’ selection proportions for each attribute among the seven tea samples were determined by Cochran’s Q test. The pairwise post-hoc McNemar test (Bonferroni) was also performed, and the tea samples signed with different alphabet letters were significantly different in the frequencies of panelists’ selection for the attribute listed on the same row. Asterisks are marked on the attributes selected by more than 20% of respondents. BLC: Bi-Luo-Chun green tea, WSP: Wenshan Pouchong tea, HMT: High-mountain oolong tea, DDO: Dong Ding oolong tea, TGY: Iron Goddess oolong tea, BHO: Baihao oolong tea and SML: Yuchi Sun-Moon-Lake black tea. “High CP (cost-performance ratio) value” could be checked when consumers felt that the tea qualities were higher than the prices they speculated.

**Table 4:** The three types of tea consumers clustered by hierarchical clustering analysis of the overall liking mean values.

Group	Overall Liking	Cluster 1	Cluster 2	Cluster 3
Percentage	109 (100%)	37 (33.9%)	49 (45.0%)	23 (21.1%)
BLC	4.71 ± 1.70 c	5.59 ± 1.64 Aa	4.82 ± 1.32 Bb	3.04 ± 1.33 Ccd
WSP	6.24 ± 1.45 a	6.27 ± 1.35 Aa	6.71 ± 1.12 Aa	5.17 ± 1.72 Bab
HMT	5.75 ± 1.50 ab	5.51 ± 1.30 ABa	6.16 ± 1.36 Aa	5.26 ± 1.86 Bab
DDO	5.91 ± 1.54 ab	5.86 ± 1.49 Aa	6.18 ± 1.35 Aa	5.39 ± 1.90 Aa
TGY	5.77 ± 1.85 ab	5.81 ± 1.93 ABa	6.20 ± 1.73 Aa	4.78 ± 1.65 Bab
HBO	5.40 ± 1.79 b	5.41 ± 1.94 Aa	6.12 ± 1.33 Aa	3.87 ± 1.46 Bbc
SML	4.02 ± 2.17 c	6.32 ± 1.45 Aa	3.16 ± 1.34 Bc	2.13 ± 1.18 Cd

There is no significant difference in consumers’ overall liking for the tea infusions marked with the same alphabet letters (*p* > 0.05). The lower-case letters are signified for comparing the data of the same column, while the upper-case letters are for the data of the identical row. BLC: Bi-Luo-Chun green tea, WSP: Wenshan Pouchong tea, HMT: High-mountain oolong tea, DDO: Dong Ding oolong tea, TGY: Iron Goddess oolong tea, BHO: Baihao oolong tea, SML: Yuchi Sun-Moon-Lake black tea.
